# Midwifery Statistics

**Published:** 1852-05

**Authors:** John Swain

**Affiliations:** Ballardsville, Ky.


ART. III.—*Midwifery Statistics.* By JOHN SWAIN, M. D., of Ballardsville, Ky.

I take the liberty to send you a table showing the number of births which have occurred within my practice during the year 1851; giving the time when the white parents were married, the present ages of both white and black, and the number of labors to each. If every physician could be induced to report like particulars to the State Medical Society, a considerable amount of very interesting matter might be deduced, of interest to the profession and the public at large. By making the minutes of each case at the time upon his day book, a physician might make out a table at the end of the year with a very little trouble. There is a Dutch notion in the country, that parturition is governed by the changes of the moon; for which reason I have noted the stage of the moon in each case to support or refute the opinion. It appears that fifteen births occurred within three days of the 1st quarter; eleven within three days of the new moon; nine within three days of the full moon; and seven within three days of the last quarter.

### A TABLE SHOWING THE NUMBER OF BIRTHS WITHIN MY PRACTICE DURING THE YEAR 1851.

| | White Male,.. | White Female, | Black Male,... | Black Female, | STAGE OF THE MOON. | Presentation,.. | When Married, | Age,........ | No. of Labour, | REMARKS. |
|---|---|---|---|---|---|---|---|---|---|---|
| January 11,.. | | 1 | | | 1st day after 1st quarte- | vertex | 1843 | 30 | 4 | |
| " 16,.. | | 1 | | | Day after full moon, | vertex | 1843 | 30 | 4 | |
| February 8,.. | | | | 1 | Day before 1st quarter. | vertex | | 20 | 3 | |
| " 17,.. | 1 | | | | 2 days after full moon. | vertex | 1843 | 20 | 6 | |
| " 27,.. | | | 1 | | 2 days before new moon. | vertex | | 16 | 1 | |
| " " .. | | | | 1 | " " " " | verter | | 19 | 2 | |
| March 1,.... | | | 1 | | 1 day before new moon. | vertex | | 27 | 6 | |
| " 4,.... | | | 1 | | 2 days after new moon. | vertex | | 21 | 1 | |
| " 20,.... | 1 | | | | 2 days after full moon. | vertex | 1831 | 42 | 9 | |
| April 6,..... | 1 | | | | 3 days before 1st quarter. | vertex | 1849 | 21 | 1 | |
| " " ..... | | | 1 | | " " " | vertex | | 20 | 2 | |
| " 8,..... | | | 1 | | 1 day before 1st quarter. | vertex | | 32 | 10 | |
| " 9,..... | | | 1 | | Day of 1st quarter. | vertex | | 36 | 6 | |
| May 10,..... | | | | 1 | 2 days after 1st quarter. | vertex | | 22 | 2 | |
| " 14,.... | | | 1 | | 1 day before full moon. | vertex | | 43 | 10 | |
| " " .... | | | 1 | | " " " | vertex | | 19 | 2 | |
| " 26,..... | 1 | | | | 2 days before new moon. | vertex | 1830 | 40 | 7 | |
| June 23,...... | 1 | | | | 2 days after last quarter. | vertex | 1839 | 31 | 5 | |
| July, 1,..... | | | | 1 | 2 days after new moon. | vertex | | 17 | 1 | |
| " 4,..... | | 1 | | | 1 day before 1st quarter. | vertex | 1830 | 38 | 9 | Could not make uterus respond to the touch, waited an hour after child was born before placenta came away. |
| " 5,..... | | 1 | | | Day of 1st quarter. | vertex | 1850 | 21 | 1 | Could not make uterus |
| " 21,..... | | | | 1 | Day of last quarter. | vertex | | 33 | 5 | respond to the touch, |
| " 31,..... | 1 | | | | 2 days after new moon. | vertex | 1833 | 34 | 9 | waited an hour for |
| August 6,... | | | | 1 | 3 days after 1st quarter- | vertex | | 25 | 5 | placenta to be thrown |
| " 29,.... | | 1 | | | 3 days after new moon. | vertex | 1840 | 25 | 7 | off. |
| September 18 | | | 1 | | Day of last quarter. | vertex | | 17 | 1 | |
| " 28 | | 1 | | | 3 days after new moon. | vertex | 1849 | 19 | 2 | |
| October, 5,.. | 1 | | | | 4 days after 1st quarter. | vertex | 1837 | 30 | 5 | Widow from '41 to '43, one child by former |
| " 11,.. | | | 1 | | 1 day after full. | vertex | | 20 | 4 | marriage. |
| " 13,.. | 1 | | | | 3 days after full. | vertex | 1846 | 30 | 4 | Wasted profusely, owing to the relax'd condition of the womb, want of contraction arrested it by applying cold water to the |
| " 18,.. | | 1 | | | 1 day after last quarter. | vertex | | 21 | 3 | abdomen. |
| " 22,.. | | 1 | | | 2 days before new moon. | dorsal v | 1836 | 20 | 1 | Labour 14 hours, water discharged before my arrival, child born im- |
| " 30,.. | | | | 1 | 1 day before 1st quarter. | vertex | 1849 | 24 | 4 | mediately after bring- |
| November 1,. | | | 1 | | 1 day after 1st quarter. | vertex | | 26 | 5 | ing down feet. |
| " 15,. | | | 1 | | 1 day before last quarter. | vertex | | 30 | 3 | Child asphyx'd; untied |
| " 17,. | | | | 1 | 1 day after last quarter. | vertex | | 24 | 2 | the chord and put in |
| December 1,. | | 1 | | | 1 day after 1st quarter. | vertex | 1837 | 30 | 4 | a warm bath. |
| " 6,. | 1 | | | | 2 days before full moon. | vertex | 1850 | '9 | 1 | Labor protracted, pains |
| " 9,. | | | 1 | | 1 day before the full moon. | vertex | | 26 | 4 | weak, amniotic liquid |
| " 17, | | | 1 | | 2 days after last quarter. | vertex | | 36 | 9 | small in quantity. |
| " 25,. | | 1 | | | 3 days after new moon. | vertex | 1833 | 38 | 9 | |
| " 31,. | | 1 | | | 1 day after 1st quarter. | vertex | 1838 | 34 | 7 | |
| | 9 | 11 | 14 | 8 | | | | | | |

